# De-regulation of gene expression and alternative splicing affects distinct cellular pathways in the aging hippocampus

**DOI:** 10.3389/fncel.2014.00373

**Published:** 2014-11-13

**Authors:** Roman M. Stilling, Eva Benito, Michael Gertig, Jonas Barth, Vincenzo Capece, Susanne Burkhardt, Stefan Bonn, Andre Fischer

**Affiliations:** ^1^Department of Psychiatry and Psychotherapy, University Medical Center GöttingenGöttingen, Germany; ^2^Research Group for Epigenetics in Neurodegenerative Diseases, German Center for Neurodegenerative Diseases (DZNE) GöttingenGöttingen, Germany; ^3^Research Group for Computational Analysis of Biological Networks, German Center for Neurodegenerative Diseases (DZNE) GöttingenGöttingen, Germany

**Keywords:** inflammaging, RNA-editing, innate immune system, RNA-seq, neuroinflammation, synaptic plasticity, learning and memory, gene-environment interaction

## Abstract

Aging is accompanied by gradually increasing impairment of cognitive abilities and constitutes the main risk factor of neurodegenerative conditions like Alzheimer's disease (AD). The underlying mechanisms are however not well understood. Here we analyze the hippocampal transcriptome of young adult mice and two groups of mice at advanced age using RNA sequencing. This approach enabled us to test differential expression of coding and non-coding transcripts, as well as differential splicing and RNA editing. We report a specific age-associated gene expression signature that is associated with major genetic risk factors for late-onset AD (LOAD). This signature is dominated by neuroinflammatory processes, specifically activation of the complement system at the level of increased gene expression, while de-regulation of neuronal plasticity appears to be mediated by compromised RNA splicing.

## Introduction

Aging is associated with a number of changes that affect cellular homeostasis and impact on the organism's overall health. Aging also leads to a decline of cognitive function including memory formation across species. As such, age-associated memory impairment is observed in invertebrates such as flies as well as in rodents and humans (Horiuchi and Saitoe, 2005; Bishop et al., 2010; Verdaguer et al., 2012). While in humans age is the most significant risk factor for neurodegenerative diseases such as Alzheimer's disease (AD), it is important to note that the degree of cognitive decline varies significantly on an individual level. Thus, some individuals undergo so-called “healthy/successful aging” that is characterized by relatively intact cognitive function, while others develop severe memory impairments and in the most extreme case dementia (Koivisto et al., 1995; Montesanto et al., 2012). In humans it is believed that the manifestation of healthy cognitive aging vs. dementia depends on the variable combinations of genetic pre-disposition and environmental factors an individual experiences throughout lifetime (Fischer, 2014). In order to decipher the molecular signature of age-associated memory impairment it is therefore most suitable to rely on mouse studies in which the genetic background and the environmental factors can be tightly controlled. The mean life span of different mouse strains housed in a laboratory ranges from about 24–30 months (Jucker and Ingram, 1997; Peleg et al., 2010). Previous studies have demonstrated that the onset of age-associated memory impairment in mice can already be observed at 16–18 months of age and is prominent at 24 months of age, while assessment of cognitive function becomes more difficult at more advanced ages due to impaired motor function (Berchtold et al., 2008; Peleg et al., 2010). It has been speculated that age-associated memory decline is correlated to a gene expression signature that dictates cellular plasticity. As such, a number of studies reported altered gene expression in the aging brain using targeted approaches such as qPCR or microarray (Finch and Morgan, 1990; Pletcher et al., 2002; Blalock et al., 2003, 2010; Lu et al., 2004; Verbitsky et al., 2004; Xu et al., 2007; Zahn et al., 2007; Loerch et al., 2008; Pawlowski et al., 2009; Bishop et al., 2010). Unlike these approaches, RNA sequencing is not biased by probe design and in addition to the identification of differential gene expression readily allows the analysis of alternative splicing and RNA editing, two processes intimately linked to cognitive function. RNA sequencing is widely used in other fields and is now also more commonly applied to study brain tissue (Dillman et al., 2013; Mazin et al., 2013; Wood et al., 2013; Stilling et al., 2014). However, the aging hippocampus, a key region for memory formation in rodents and humans that is affected early in age-associated memory decline and AD, has not been studied using RNA sequencing. To this end, we used Illumina next-generation sequencing to compare the hippocampal transcriptomes of 3, 24, and 29-month-old C57BL/6J mice (*3M, 24M*, and *29M*, respectively). We find that the aging hippocampus is characterized by a strong neuroinflammatory gene-expression signature that is dominated by differential gene expression but not by differential splicing or RNA-editing. A key component of the neuroinflammatory response was activation of the complement system that has repeatedly been genetically linked to AD (Bertram et al., 2007; Lambert et al., 2009; Brouwers et al., 2012). Taking into account that neuroinflammation is a key mechanism in neurodegenerative diseases, our data supports the view that AD may represent accelerated brain aging due to an unfavorable genetic pre-disposition and exposure to environmental risk factors. On the other hand, we find that compromised synaptic function is linked predominantly to alternative splicing suggesting that, on the level of the transcriptome, age-associated neuroinflammation and decreased synaptic plasticity are mediated by distinct cellular processes.

## Materials and methods

### Animals

Specific pathogen free (SPF) C57Bl6/J wild type mice were obtained from Janvier SAS. Mice were kept in groups ≤5 animals in individually ventilated cages (32 × 16 × 14 cm, Techniplast) on a 12 h light/dark cycle with food and water *ad libitum*. To obtain mice between 28 and 30 months of age, 24 month-old mice were ordered from Janvier SAS and kept in our holding rooms for 4–6 months. All procedures were performed by experienced experimenters and according to protocols approved by the Lower Saxony State Office for Consumer Protection and Food Safety.

### Novel object recognition

Behavioral testing was performed as described previously (Kerimoglu et al., 2013). Animals were habituated individually to a uniform-gray plastic arena (90 × 90 cm, walls 20 cm high) for 5 min on two subsequent days. Animals were then further habituated to two equal objects placed in opposing corners of the arena for 5 min on the next two days. On day 5 objects were exchanged by two new but equal objects (A + A) and animals were allowed to explore the objects for 5 min. Then, mice were sent back to their home cages for 5 min (for short-term memory assessment) and reintroduced to the arena after one object was exchanged (objects A + B). After 24 h, object B was exchanged for object C for long-term memory assessment. Duration of object contacts was measured. Mice that only showed summed contact time of <1 s were excluded from the analysis of this test. Object preference was defined as (novel object)/sum(both objects).

### RNA extraction and sequencing

Total RNA was extracted using TRI Reagent (Sigma-Aldrich) as previously described (Peleg et al., 2010). In brief, flash-frozen tissue was homogenized on ice with several pestle strokes in 0.5 ml of TRI Reagent (Sigma-Aldrich) After addition of another 0.5 ml of TRI Reagent and 5 min incubation at room temperature (RT) the dissociated homogenate was mixed with 300 μl of CHCl3 and incubated for 15 min (RT) followed by centrifugation at 12,000× g (4°C). The upper aqueous phase was transferred to a new tube, mixed with 500 μl isopropanol and incubated at -20°C for at least 1 h for precipitation. RNA was precipitated by centrifugation at 12,000 g for 30 min (4°C). The pellet was washed twice with 1 ml of 75% ethanol (centrifugation after washing steps: 12,000× g, 5 min, 4°C). The washed pellet was dissolved in 30 μl of RNase-free water. Following DNase1 (life Technologies) treatment to remove residual contaminating genomic DNA for 20 min at 37°C, RNA was purified using phenol-chloroform extraction. Library preparation and cluster generation for mRNA sequencing [single-end libraries for 3M (*n* = 5) vs. 24M (*n* = 6) comparison; paired-end libraries for 3M vs. 29M comparison (*n* = 3)] was performed according to Illumina standard protocols using the TruSeq RNA Sample Prep Kit v2 and the TruSeq Paired-End Cluster Generation Kit v3-cBot-HS (for paired-end mRNA-seq) with subsequent use of the corresponding TruSeq Cluster Generation Kit v3-cBot-HS (for single-end mRNA-seq). Libraries were quality controlled and quantified using a Nanodrop 2000 (Thermo Scientific), an Agilent 2100 Bioanalyzer (Agilent Technologies) and Qubit (life Technologies). For the sequencing run, TrueSeq SBS kits were used according to Illumina manuals. Read lengths were 1 × 50 bp for single-end and 2 × 100 bp for paired-end sequencing.

### Bioinformatic analysis pipeline

#### Differential gene expression

Differential gene expression analysis of RNA sequencing data was performed as described previously (Stilling et al., 2014). In brief, library preparation [single-end libraries for 3-month (3M) (*n* = 5) vs. 24-month (24M) (*n* = 6) comparison; paired-end libraries for 3M vs. 29-month (29M) comparison (*n* = 3)] and cluster generation for mRNA sequencing were performed as required by Illumina protocols (TruSeq, Illumina). Downstream analysis steps after read retrieval included quality control (FastQC, www.bioinformatics.babraham.ac.uk/projects/fastqc/) and mapping to reference genome (STAR aligner v2.3.0, Dobin et al., 2013). For calling of differentially expressed genes (DEG), mapped reads were counted with HTSeq v0.5.4p2 (http://www-huber.embl.de/users/anders/HTSeq) (non-default parameters: -m intersection non-empty) and count tables were analyzed independently for both aging groups vs. the 3M young control group using the DESeq2 v1.2.5 R-package (Anders and Huber, 2010). Genes with a log_2_(fold-change) ≥ 0.5 and adjusted *p*-value ≤ 0.05 were considered differentially regulated. All expression data are made publicly available in a GEO SuperSeries (GSE61918). Compressed fastq-files and primary analysis can be found under GEO accession GSE61915, microarray raw data is available under GEO accession GSE61647.

#### Functional annotation

Functional annotation and category and pathway analysis was carried out using the Database for Annotation, Visualization, and Integrated Discovery (DAVID, v6.7) (Huang et al., 2009a,b). Non-cutoff-based gene set enrichment analysis (GSEA) was performed by using the Broad Institute GSEA application (Mootha et al., 2003; Subramanian et al., 2005). Input lists for GSEA were ranked based on log_2_-fold-change.

#### Transcription factor binding sites

Promoter analysis to search for overrepresented transcription factor binding sites (TFBS) was done using the Pscan web interface (Zambelli et al., 2009) (http://159.149.160.51/pscan/) scanning the promoter region from −250bp to +50bp from TSS comparing to JASPAR descriptors. All Gene ID conversion was done using BioMart database queries (www.ensembl.org/biomart/).

#### Differential exon usage

For detection of differential exon usage the DEXSeq R-package was used (Anders et al., 2012) with default parameters. FDR-corrected *p*-value significance level was set to 0.01 for the 3M vs. 24M comparison and to 0.1 for the 3M vs. 29M comparison to adjust for differences in library-type and sample size.

#### RNA-editing

Known RNA-editing positions in the mouse reference genome (mm10/GRCm38 coordinates) were retrieved from previously described editing sites in the mouse genome from two online databases and two recent publications and compiled to a non-redundant list of 17831 positions (Table S6). This list was used as input for the REDItools algorithm REDItoolKnown.py (Picardi and Pesole, 2013). Further input arguments were a list of splice sites (taken from UCSC table browser), the current genome sequence (GRCm38p2) in fasta format and the most recent GTF file (taken from Ensembl, version GRCm38.e75). Non-default parameters were –C 1000, –c 0, –q 10, –m 10, –v 1, –n 0.001, –t 4. For statistical comparison between groups independent, two-sided Student's *t*-tests were used for each position.

### Microarray

RNA quality control, cDNA synthesis, mono-color Cy3-labeling and hybridization to whole mouse genome microarray chips were carried out according to Standard Operating Procedures of the Transcriptome Core facility at University of Göttingen. Total RNA was labeled with Cy3 according to Agilent's Low RNA Input Fluorescent Linear Amplification Kit and later hybridized to Agilent Whole Mouse Genome 4 × 44K G4122F microarrays according to the manufacturer's protocol. Quantity and Cy3-dye incorporation rates of the generated target material were assessed using a NanoDrop ND-1000. Washes were performed according to the Agilent Technologies SSPE protocol (v2.1)—wash solution 3 was replaced by acetonitrile. After that, scanning was performed using an Agilent G2505B scanner. Data analysis was performed as described previously (Peleg et al., 2010; Agis-Balboa et al., 2011; Kerimoglu et al., 2013). In summary, data was analyzed using Agilent Feature Extraction software, version 9.5.3.1 and the Limma (Smyth et al., 2005) package for R/Bioconductor (Gentleman et al., 2004). In order to assure that the intensities had similar distributions across arrays, VSN normalization (Huber et al., 2002) was applied to the intensity values as a method for between-array normalization. To estimate the average group values for each gene and assess differential gene expression, a simple linear model was fit to the data, and group-value averages and standard deviations for each gene were obtained. To find genes with significant expression changes between groups, empirical Bayes statistics were applied to the data by moderating the standard errors of the estimated values (Smyth, 2004). *P*-values were inferred from the moderated t-statistic and corrected for multiple testing using the FDR method (Benjamini and Hochberg, 1995). Afterwards, the final output was filtered for probes showing a change in normalized intensity that was greater than 1.414 – fold [log_2_(fold change) ≥ 0.5] with an adjusted *p*-value of FDR(p)<0.1.

### Quantitative real-time PCR (qRT-PCR)

Quantitative real-time PCR (qPCR) was performed as described before (Peleg et al., 2010). In summary, 1 μg of total RNA was used for cDNA synthesis and cDNA was diluted 1:10. Probe-based qRT-PCR (UPL, Roche) was carried out on a LightCycler 480 II (Roche) and analyzed using suppliers software. Primers used for *C4b* amplification: Fwd(5′-TCTCACAAACCCCTCGACAT-3′), Rev(5′- AGCATCCTGGAACACCTGAA-3′), UPL-Probe #10.

### Immunohistochemistry

Fluorescent staining of target proteins was performed as previously described (Peleg et al., 2010). In brief, mice were transcardially perfused with 4% PFA, brains isolated and post-fixed for another 16 h in 4% PFA. Free-floating cryosections (30 μm) were incubated with 5% goat serum for blocking and followed by incubation with target-specific primary antibodies (anti-NeuN [A60, MAB377, Merck Millipore, 1:1000, anti-GFAP [G5601, Promega, 1:1000], anti-IBA1 [019-19741, WAKO, 1:1000]). Corresponding secondary antibodies were from life Technologies (anti-mouse Alexa-488 labeled, A11029; anti-rabbit Alexa-633 labeled, A21071). Images were taken on a Leica SP2 confocal microscope. Stereological analysis of the number of cells was performed on 4 serial 40 μm free-floating coronal sections per animal which were analyzed by confocal microscopy to count cells expressing the indicated marker. Cell number was assessed as areal density across the CA1 region. The data was normalized to the 3 month groups.

## Results

### Differential gene expression analysis in the aging hippocampus

**Figure 1:**
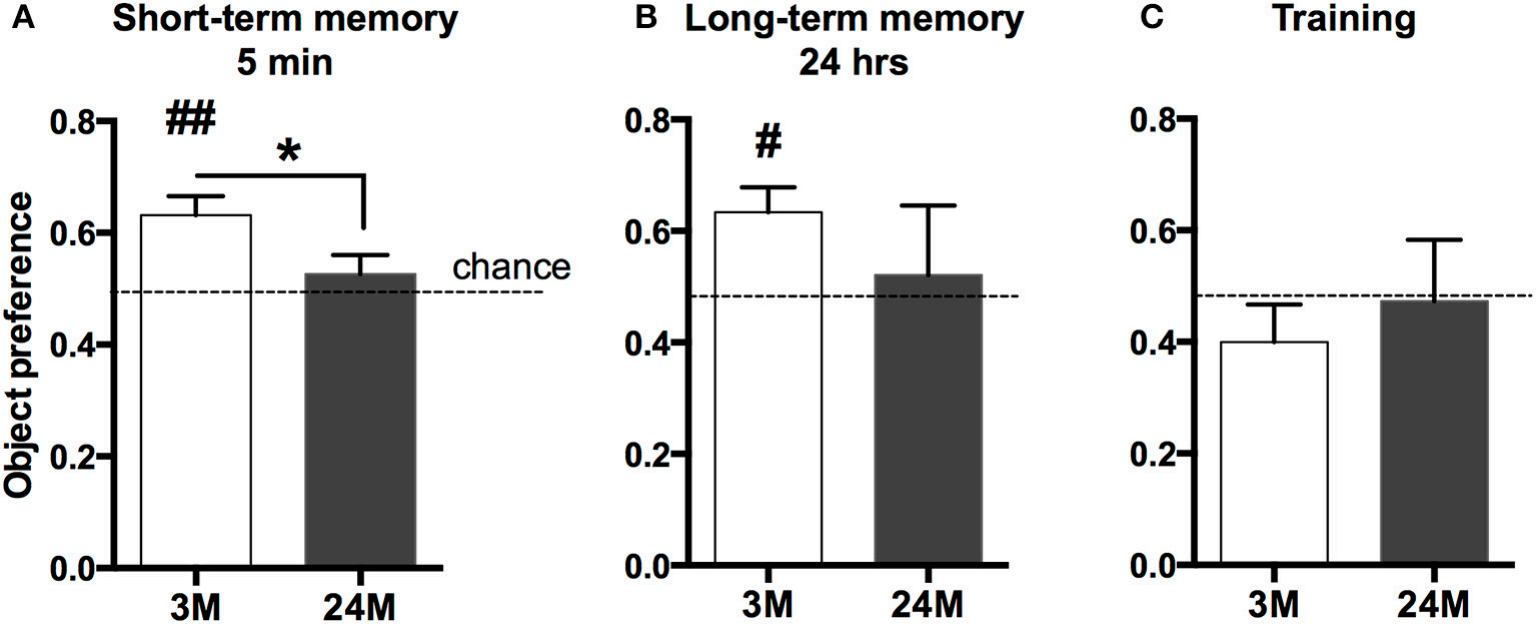
**Impaired novel object recognition memory in 24-month-old mice**. **(A)** 3-month-old mice (3M) and 24-month-old mice (24M) were tested in the novel object recognition paradigm. To assay short-term memory, mice were first exposed to two similar objects (A+A) and then re-exposed to the testing area containing one novel object 5 min later (A+B). Preference for the novel object was significantly greater in 3-month-old mice when compared to the 24-month group, which performed at chance level (^*^p < 0.05 between groups, two-sided *t*-test; ^##^*p* < 0.01 vs. chance level, one-sample *t*-test; *n* = 11[3M]/9[24M]). **(B)** To test long-term memory mice were exposed to the arena 24 h later, now containing another novel object (A + C). While object preference in 3M was significantly higher than chance level (^#^*p* < 0.05, one-sample *t*-test, *n* = 10), 24M animals (*n* = 4) performed at chance level, indicating impaired long-term object recognition memory in aged mice. Error bars indicate s.e.m. **(C)** During the training session, none of the groups showed preference for any of the two equal objects (A+A).

Age-associated memory impairment is the result of variable combinations of genetic pre-disposition and environmental factors, which eventually causes detrimental changes in cellular homeostasis. We therefore reasoned that a comprehensive picture of age-related changes in transcription would be most informative about the aging processes occurring in the brain. The hippocampal formation is essential for memory function in rodents and humans and has been linked to cognitive age-associated memory impairment (Fanselow, 2010). Thus, we performed deep sequencing of polyA-enriched RNA extracted from the mouse hippocampus in three different age groups (3-month-old mice, 24-month-old-mice, and 29-month-old-mice). Since C57BL/6J mice in the laboratory have a maximum life span of just above 30 months, 24-month-old mice represent a model of advanced aging, while 29-month-old represent a time point at the end of life-span. We first confirmed that the selected groups of mice indeed show age-associated memory decline (Figure 1). Due to severely impaired locomotor activity in 29-month-old mice we had to exclude these mice from any behavioral testing. A commonly employed test for hippocampus-dependent memory function in mice is the Morris water maze test. Pilot experiments however showed that in our hands even 24-month-old mice have difficulties to cope with the 2-week-lasting daily training procedure, in which the animals have to swim in a pool filled with opaque water and need to find and climb on a hidden platform. Thus, we decided to subject mice to the novel-object-recognition paradigm that does not depend on advanced motor skills and allows the measurement of short and long-term memory in a non-stressful experimental setting. Moreover, while object recognition learning recruits various brain structures, it also depends on an intact hippocampus (Broadbent et al., 2010; Antunes and Biala, 2012). As expected, we observed that both short (Figure 1A) and long-term object-recognition memory (Figure 1B) was impaired in 24-month-old mice, when compared to 3-month-old mice. This was not due to altered explorative behavior during the training, since both groups of mice explored the objects presented during the training session to a similar degree (Figure 1C).

**Figure 2:**
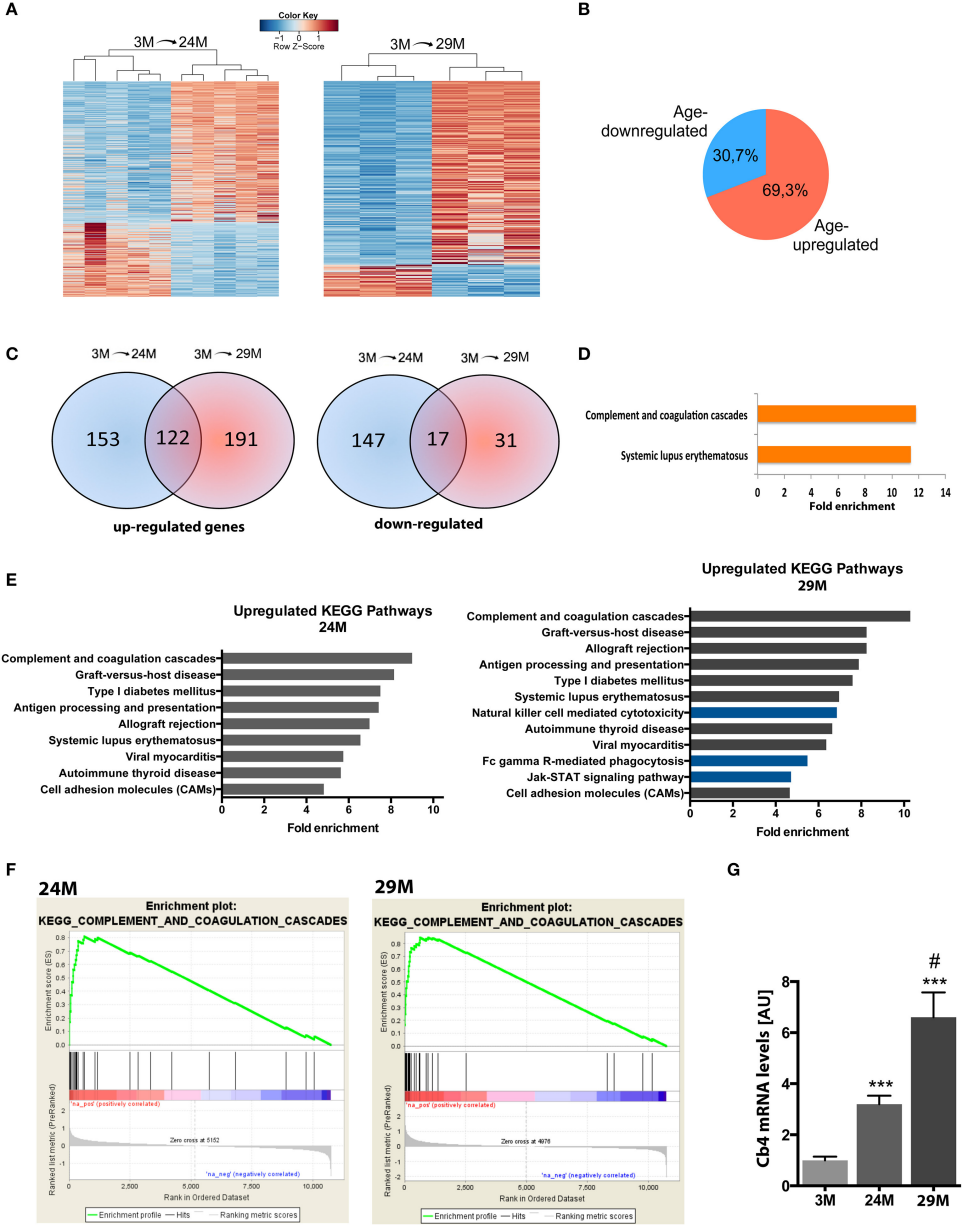
**Aging is associated with changes in gene expression and upregulation of immune system functions in the hippocampus. (A)** Heatmaps of differentially expressed genes in 3-month (3M) and 24-month-old mice (24M, left panel) and 3 and 29-month (29M) old mice (right panel). High expression is marked by the red color spectrum, low expression by blue colors. **(B)** Pie chart showing the percentage of genes up-and down-regulated in 3 vs. 24/29 month-old mice. **(C)** Venn diagrams comparing significantly regulated genes found in the different experiments (3M vs. 24M and 3M vs. 29M). **(D)** Genes up-regulated at 24 and 29 month of age were subjected to functional enrichment analysis for overrepresented biological processes and KEGG pathways. **(E)** Fold enrichment of overrepresented KEGG pathways in up-regulated genes when individually comparing the 3M vs. 24M and 3M vs. 29M groups. **(F)** Using non-cutoff gene set enrichment analysis (GSEA), the top up-regulated KEGG pathway was the complement and coagulation cascade. Shown are enrichment plots along expressed genes, ranked by fold-change [left/red: positive log_2-_fold-change (upregulation), right/blue: negative log_2_-fold-change (downregulation); a gene contributing to the enrichment score of the selected pathway along the ranked list is marked by a vertical black line and increases the cumulative enrichment score (green line)]. **(G)** qPCR analysis of the *C4b* gene (^***^p < 0.001 vs. 3M group, two-sided *t*-tests; ^#^*p* < 0.05 vs. 24M group, two-sided *t*-test; *n* = 5[3M]/4[24M]/8[29M]). Error bars indicate s.e.m.

In the next step, we isolated total RNA from the hippocampi of 3-, 24-, and 29-month-old mice and subjected it to RNA sequencing. In line with previous studies, we observed that aging was not associated with massive changes in cell number (Long et al., 1999) (Figure S1). We first compared differential gene expression across the different age groups using the 3-month group as reference. 477 genes were differentially expressed (313 up-regulated, 164 down-regulated) in 24-month-old mice and 323 genes (275 up-regulated, 48 down-regulated) in 29-month-old mice (Figure 2A, Table S1) when compared to the 3-month group. In all comparisons, we observed a general trend toward higher numbers of up-regulated genes (~70%) compared to down-regulated genes (Figure 2B). When we compared these lists among each other, we found a significant amount of overlap between genes up-regulated at 24 and 29 months of age (122 genes, Figure 2C, Table S1). The overlap between the genes down-regulated in 24- and 29-month-old mice was less pronounced (17 genes, Figure 2C, Table S1). When we analyzed the 122 genes commonly up-regulated in 24- and 29-month-old mice for functional pathways we observed inflammatory signaling pathways, namely the “Systemic lupus erythematosus” and the “complement and coagulation pathway” to be highly enriched (Figure 2D, Table S2). It has to be noted that several genes of the complement system are linked to systemic lupus erythematosus, which explains the enrichment of this pathway (Table S2). This data suggests that activation of the complement system is one of the key features of the aging hippocampus. Similar results were obtained when we separately analyzed all genes up-regulated in 3 vs. 24-month-old mice (Figure 2E, left panel). When we analyzed all up-regulated genes in 3 vs. 29-month-old mice, we found significant enrichment of additional immune-related pathways (Figure 2E, right panel, blue bars), suggestive of an even more pronounced immune activation with increasing age. Another group of up-regulated genes was associated with cell adhesion since “cell adhesion molecules” was also identified as a significantly enriched pathway in 24- and 29-month-old mice (Figure 2E, Table S2).

These findings, based on analysis of lists of significantly up-regulated genes using the DAVID platform, were further confirmed by GSEA, an unbiased approach to analyze enrichment of functional groups in a given gene list without the need for thresholding or introduction of *p*-value cutoffs. Similar to the previous analysis, we found the enriched immune system pathways, including the top-enriched “complement and coagulation pathway” in both aging groups (Figure 2F), as well as several additional pathways associated with immune-system function (Figure S2). Interestingly, several of these additional categories were associated with pathological infection by bacteria, viruses and other pathogenic organisms, suggesting that at least part of the neuroinflammatory response may be attributed to the invasion of parasitic microbes across the blood brain barrier. Furthermore, GSEA found a similar set of additional pathways in the 29-month-old mice compared to the 24-month group as detected by DAVID platform analysis. Thus, GSEA confirmed functional enrichment of immune system-related pathways, which are gradually up-regulated with advancing age.

To further verify these findings we decided to reproduce the results in an additional cohort of 3 and 29-month-old mice using a microarray approach thereby also controlling for any potential experimental bias that might be introduced by RNA sequencing. Albeit DEG differed to some extend, the affected pathways were almost identical and the complement system was the most affected pathway among the up-regulated genes (Table S2).

Within this pathway the *C4b* gene encoding the complement factor 4 (C4) constantly turned up among the most significantly up-regulated genes in all our analyses. Thus, to validate the finding made by genome-wide techniques we sought to directly compare expression levels of this gene among all three aging groups by qRT-PCR. We detected a 3.2-fold increase of *C4b* mRNA levels in the 24-month (24M) group and a further increase in 29-month (29M) group (6.6-fold) when compared to the 3-month (3M) group (Figure 2G).

We also determined functional enrichment among down-regulated genes. Interestingly, the down-regulated genes in the 24M group were enriched for genes of the “neuroactive ligand-receptor interaction” and “regulation of transcription” pathways, while no pathways could be detected in the 29M group, even if we combined the data obtained by RNA sequencing and microarray (Table S2). When we combined the genes down-regulated in 24- and 29-month-old mice we observed the “neuroactive ligand-receptor interaction” and the “calcium signaling” pathways to be significantly affected (Table S2).

In addition to protein-coding genes, we could also detect differential regulation of several non-coding RNAs (ncRNAs), including the pseudogene *Pisd-ps1* and the long intergenic non-coding RNA (lincRNA) *Neat1* as well as *Malat1*, also known as *Neat2* (Table S1). While the role of long non-coding RNAs is only beginning to emerge, *Neat1* and *Malat1* are known to accumulate in the nucleus, where they form the RNA backbone of so-called paraspeckles, subnuclear ribonucleoprotein bodies involved in transcriptional regulation, e.g., by nuclear retention of RNAs (Bond and Fox, 2009). Interestingly, we could also detect increased transcription from the *C4a* locus in the 29M group. *C4a* is closely related to the nearby *C4b* but does not encode a protein, according to the ENSEMBL database. Though their functional significance is subject to ongoing research, these results indicate that also regulatory ncRNAs are implicated in hippocampal aging.

Taken together, analysis of differential gene expression revealed that hippocampal aging is markedly characterized by up-regulation of a neuroinflammatory program. Especially activation of the complement system was highly correlated with increasing age.

### Age-associated changes in gene expression are orchestrated by a specific set of transcriptions factors

**Figure 3:**
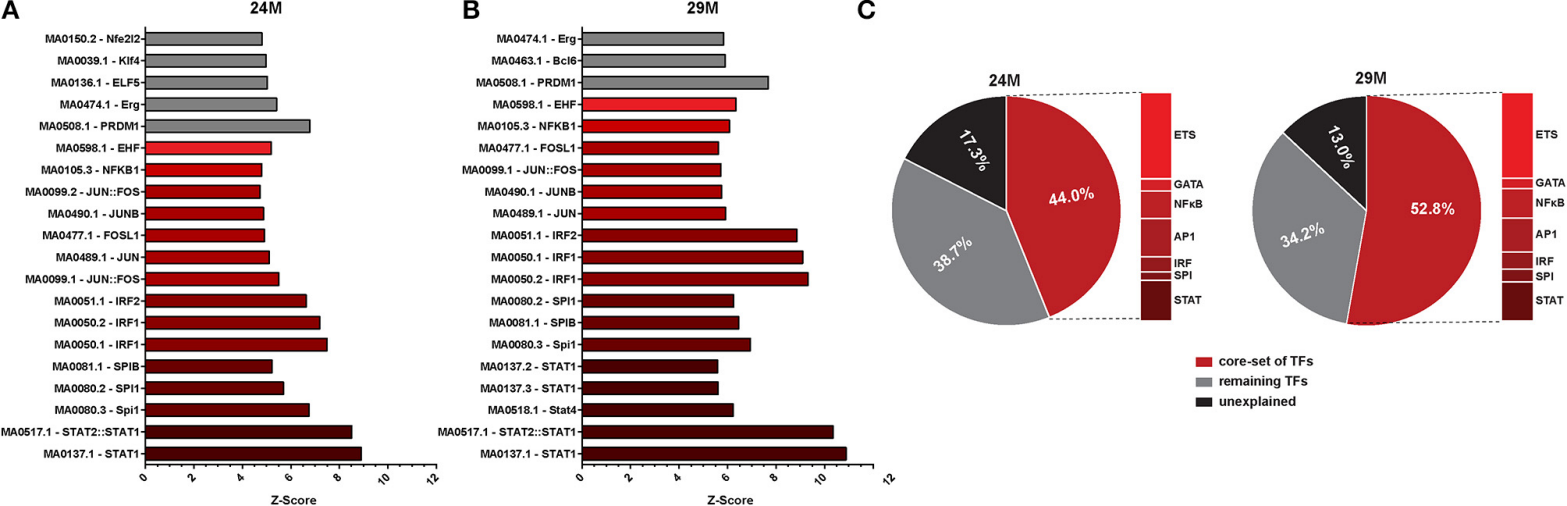
**Promoter analysis of aging-regulated genes for overrepresented transcription factor binding sites (TFBS). (A)** Z-score (enrichments score) for different position weight matrices of known transcription factors in the set of gene up-regulated in 3 vs. 24-month-old mice. Higher Z-score means higher enrichment. Shown are only the top 20 most significantly enriched matrices. **(B)** The same analysis as described in **(A)** was performed for up-regulated genes in 3 vs. 29-month-old mice. **(C)** Pie charts showing the percentage of differentially expressed genes that can be explained by the selected core set of transcription factors.

To further elucidate potential upstream mechanisms of the identified transcriptional program, we searched the promoters of regulated genes for common TFBS. We found a large number of potential TFBS significantly enriched at promoters of genes that were up-regulated with aging (Table S3). Of these, the TOP20 most strongly enriched TFBS were largely similar in the 24M and 29M group (Figures 3A,B). We could identify a number of common transcription factor families that together made up the bulk of the significantly enriched TFBS [*Signal transducer and activator of transcription* (STATs), *Interferon regulatory factor* (IRF), *Spleen focus forming virus proviral integration oncogene* (SPI), Activator protein 1, (AP1, composed of Fos, Jun and ATF family members), *Nuclear factor kappa-light-chain-enhancer of activated B cells* (NF-κ B), the GATA and the *E26 transformation-specific* (ETS) family of transcription factors (Table S3)], which are strongly associated with their roles in immune-related signaling (Peng, 2008). Of note, this set of TFs explained 44% of all up-regulated genes in 24- and 53% in 29-month-old mice. In fact, up to 87% of all up-regulated genes could be assigned to the action of enriched TFs (Figure 3C, Table S3).

In agreement with functional enrichment of DEG, we observed a higher diversity of enriched TFBS in the 29M group (Figure 3B, Table S3). While TFBS of the AP1 family were more abundant in the 24M group, TFBS of the NF-κ B and GATA families were more abundant in the 29-month group. Notably, also the Z-scores and significance levels for the STAT and IRF families were considerably higher in the 29M group (Figures 3A,B Table S3), which was in line with up-regulation of the *Stat1* gene and several *Irf* genes in the 29M group (Table S1). Together, these results suggest increasing usage of STAT- and IRF-related cellular signaling pathways in the hippocampus with increasing age.

Among down-regulated genes, significant enrichment of TFBS was less pronounced and more heterogeneous. However, binding sites for the E2F family of transcription factors as well as for the specificity protein (SP) and EGR families were identified as common between the two aging groups and enrichment of EGR TFBS among down-regulated genes correlated with down-regulation of *Egr* genes in the 24-month group (Tables S1, S3).

### Widespread and specific alternative exon usage changes in the aging hippocampus

Alternative to the altered expression of genes, co-transcriptional intron excision from the nascent pre-mRNA, known as splicing, is the main mechanism for generation of transcript isoforms and differential exon use in information-dense and complex genomes, thereby adding another important level of gene expression control.

**Figure 4:**
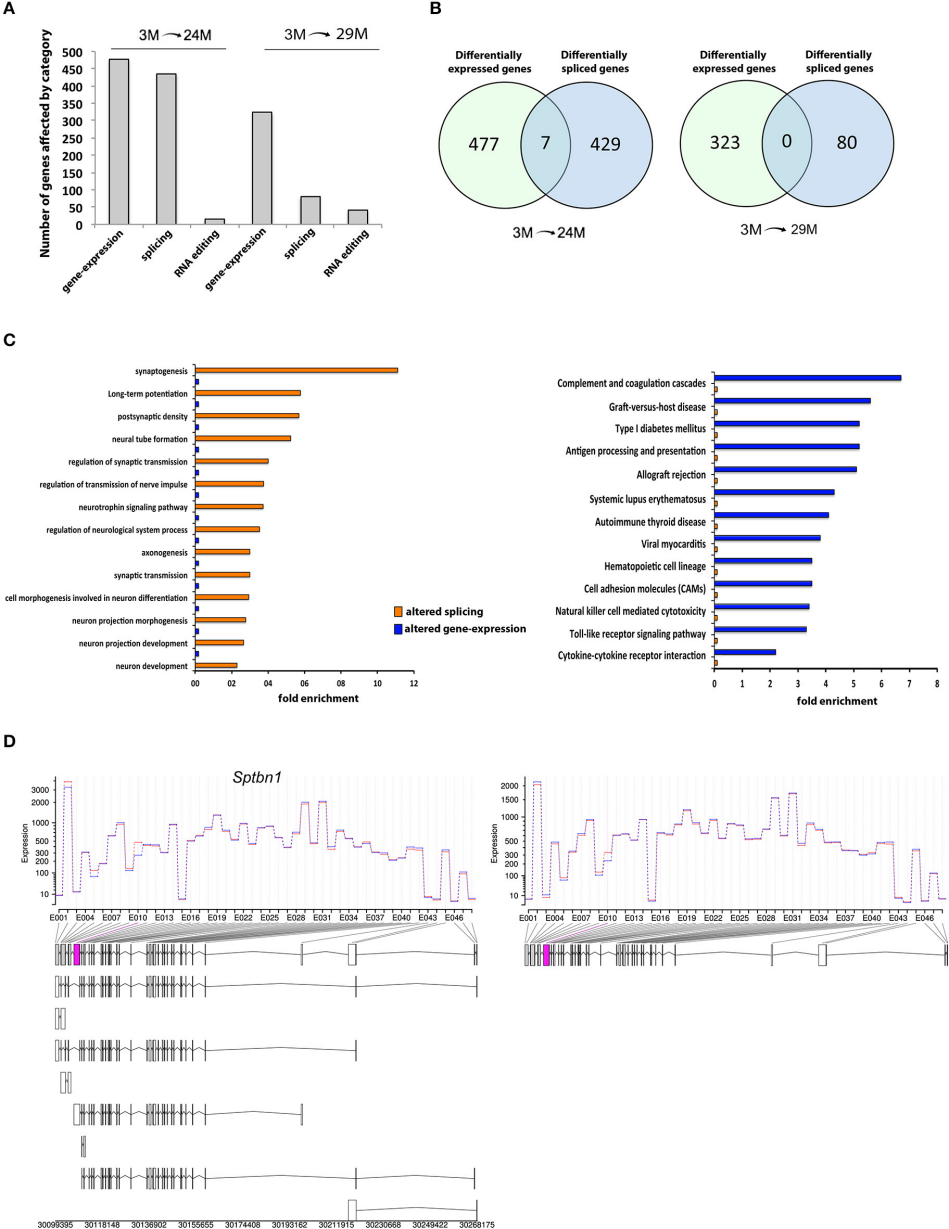
**Differential exon usage and alternative splicing changes. (A)** Number of hippocampal genes affected by differential expression, splicing or RNA-editing during aging. **(B)** Venn-diagrams showing the overlap of genes affected by expression and/or splicing between age groups. Note that there is little to no overlap. **(C)** Cellular pathways affected in 3- vs. 24/29-month-old mice by splicing or expression levels. The data on differential expression is based on all up- or down-regulated genes. **(D)** One of the overlapping genes was *Spectrin β, non-erythrocytic 1 (Sptbn1)*, showing higher expression of exon 10 (pink box) in aged mice, suggesting higher abundance of the transcript isoform 2 (see Figure S2 for details on isoform and domain structure).

We therefore analyzed our RNA sequencing data with respect to differential exon usage. We found 436 annotated genes with significant changes in exon usage in the 24M group and 80 genes in the 29M group (Figure 4A; Table S4). Thus, changes in RNA splicing were, at least in the 24M group, quantitatively comparable to the changes observed in gene expression (Figure 4A). However, there was little to no overlap between genes affected by altered splicing and genes that were differentially expressed (Figure 4B). In fact, less than 1% of the DEG were also differently spliced in the 24M group, while zero overlap was found in the 29M group. This data indicates that differential gene expression and alternative splicing may affect different signaling pathways. To test this hypothesis, we analyzed the differentially spliced genes for enrichment of functional pathways. Our analysis revealed that there was a significant overrepresentation of genes associated with neuronal function including synaptogenesis, regulation of synaptic transmission, axonogenesis, neuron projection morphogenesis, postsynaptic density and long-term potentiation (Figure 4C, Table S5). Of note, none of these pathways was enriched in gene-set of DEG (Figure 4C). Vice versa, pathways linked to inflammatory response–which dominated the list of DEG–could not be identified within the group of alternatively spliced genes (Figure 4C). These data suggest that inflammatory responses in the aging hippocampus are driven by changes in differential gene expression whereas de-regulation of synaptic plasticity in mainly attributed to differential splicing. One of the genes that was differentially spliced in both 24- and 29-month-old mice when compared to their young counterparts was the *Spectrin β, non-erythrocytic 1* gene (*Sptbn1*), that showed a specific upregulation in usage of exon 10 with age (Figure 4D). *Sptbn1* is best known for its role in cytoskeleton regulation during neurite outgrowth (Lee et al., 2012), which is in agreement with our previous functional enrichment analysis. The inclusion of exon 10 suggests a shift towards higher expression of *Sptbn1* isoform 2, resulting in a protein that has shorter and distinct N- and C- termini compared to isoform 1 and lacks the pleckstrin homology (PH) domain that is critical for tethering F-actin filaments to the plasma membrane (Figure S3).

In addition to these widespread changes, we found differential exon usage in the *amyloid beta (A4) precursor protein* (*App*) and the *beta-site APP cleaving enzyme 1* (*Bace1*) genes (Table S4), both of which are strongly implicated in the etiology of AD. This suggests that also differential-splicing events that occur with aging may be implicated with altered Aβ production in AD.

### Differences in RNA-editing

A second mechanism to generate alternative transcript isoforms is RNA-editing. Though more subtly changing the coded information as compared to alternative splicing, it also occurs co-transcriptionally and may lead to altered regulation of the transcript or a change in amino acid sequence of the encoded protein. Notably, the mammalian, and especially the human, brain has been identified as the principal site for RNA-editing. Since RNA sequencing is not only able to quantify transcript abundance but also allows visualization of the exact sequence of these transcripts, it is possible to identify changes in transcript sequence from the genomic reference at known positions. We compiled a list of previously described editing sites in the mouse genome from two online databases and two recent publications to yield a non-redundant list of 17831 positions (Table S6).

**Figure 5:**
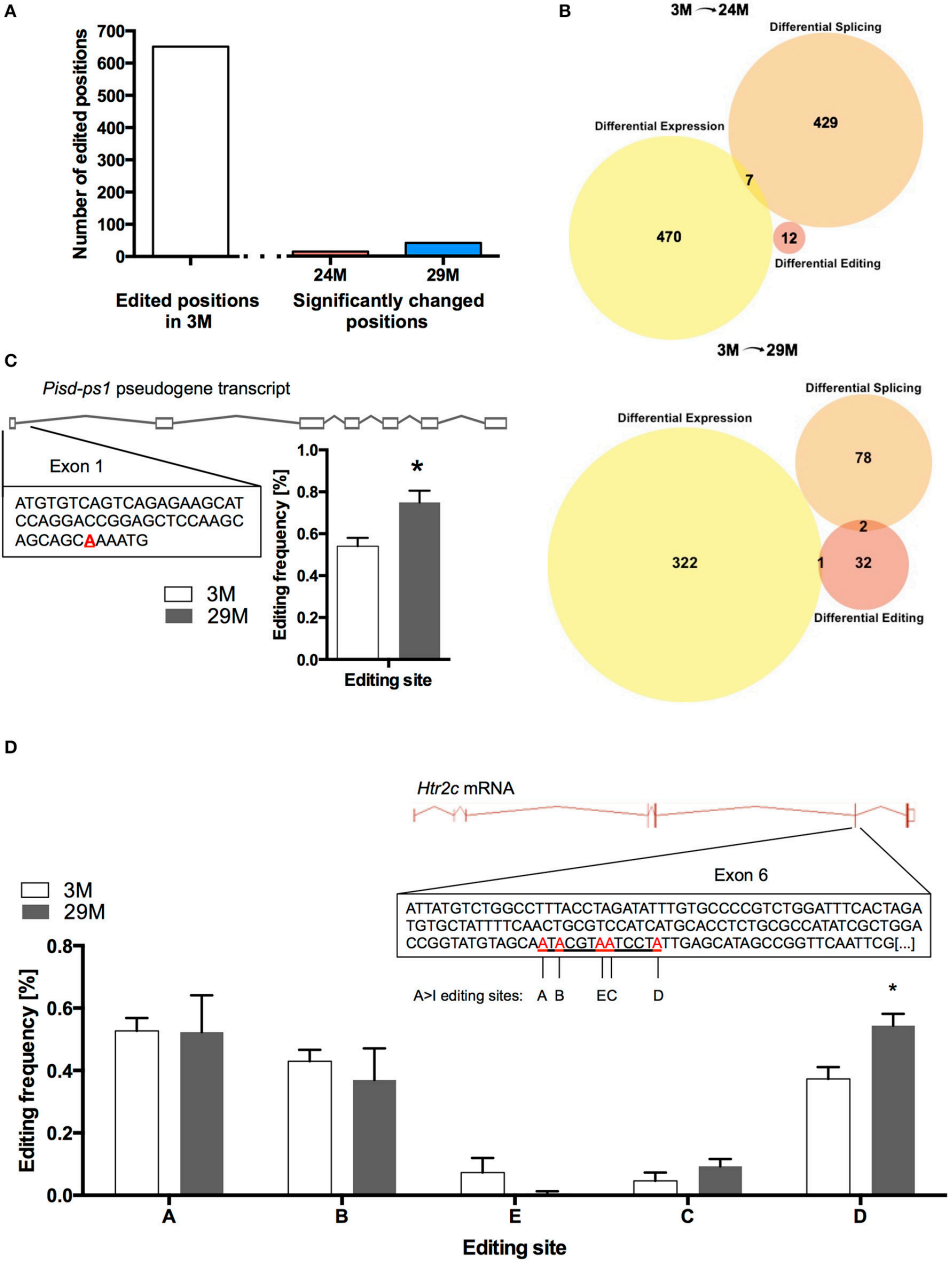
**RNA editing in the aging hippocampus**. **(A)** Total number of RNA-editing events detectable in the hippocampus of 3-month-old mice and number of changes occurring during aging. **(B)** Venn diagram showing the overlap of differentially expressed genes, genes affected by splicing and RNA-editing in 24-month-old mice (upper panel) and 29-month-old mice (lower panel). **(C)** Editing of the pseudogene-transcribed ncRNA *Pisd-ps1* within exon 1 was found to be significantly increased in the 29M group **(D)** Within the well-described editing cassette of the *Htr2c* mRNA, editing was significantly upregulated at position D (^*^p < 0.05, *n* = 3 per group). Error bars indicate s.e.m.

At 682 of the 17831 known positions, we found RNA-editing in the 3M group (Table S7). Of these 682 positions, we found 14 editing sites in the 24M group and 41 editing sites in 29M that showed significant change in editing frequency, corresponding to 12 and 35 genes, respectively (Figure 5A, Table S8). Interestingly, when we compared genes that undergo altered RNA editing in the aging hippocampus to the list of differentially expressed and spliced genes, there was almost no overlap (Figure 5B) suggesting that gene expression, splicing and RNA editing control distinct cellular pathways in the aging hippocampus. Only one of the genes that was characterized by altered RNA editing in 29-month-old mice was also up-regulated at the gene expression level (Figure 5C). This gene (*Pisd-ps1)* codes for a non-coding RNA expressed from a pseudogene with unknown function.

We also found increased editing of the well-established RNA-editing target *serotonin receptor 2c* (*Htr2c*) (Figure 5D). It has been shown that increased editing of the *Htr2c* mRNA leads to a decrease in the receptor's signaling fidelity, which is in line with declining function of the serotonergic system with aging (Fidalgo et al., 2013). Moreover, altered RNA-editing of the Htr2c gene was observed in a mouse model for impaired memory function (Stilling et al., 2014).

Another gene that showed higher editing frequency with aging was *Slc7a2*, also known as *Cat2* or *Ctn*. Interestingly, it has been described in the literature that the *Ctn* isoform of this gene undergoes nuclear retention and becomes part of paraspeckles (Bond and Fox, 2009), supporting the previous observation that aging is accompanied by increased nuclear paraspeckle formation.

In summary, our results suggest that hippocampal aging correlates to different degrees with altered gene expression, RNA splicing and RNA editing. Of note all of these processes appear to specifically account for age-associated changes in distinct cellular pathways. Namely, the induction of inflammatory processes is linked to altered gene expression while decline of synaptic plasticity seems to be mainly due to differential splicing.

## Discussion

In the present study we performed RNA sequencing in the hippocampus of three different aging groups to determine differential gene expression, exon usage and RNA-editing. Though brain aging has previously been described to go along with transcriptional changes, a detailed, homogenous picture of the transcriptome of the aging mouse hippocampus, especially toward the end of an individual's life span using RNA sequencing, has not been drawn yet.

Several studies have used microarray approaches to investigate age-associated transcriptional profiles of different brain regions in different species and came to heterogeneous results (Finch and Morgan, 1990; Pletcher et al., 2002; Blalock et al., 2003, 2010; Lu et al., 2004; Verbitsky et al., 2004; Xu et al., 2007; Zahn et al., 2007; Loerch et al., 2008; Pawlowski et al., 2009; Bishop et al., 2010), which may in part reflect differences in the technology available and also limited information on the mouse genome and transcriptome at the time the corresponding studies were conducted.

When we analyzed differential gene expression among the different age groups, the most striking effect was the up-regulation of genes linked to inflammatory processes with the complement system being the top ranking pathway identified by gene-set enrichment, as well as pathway analysis using the Kyoto Encyclopedia of Genes and Genomes (KEGG) database. Increased neuroinflammation is likely the result of multiple up-stream factors such as impaired blood-brain barrier function (Lynch and Johnson, 2012; Erickson and Banks, 2013) leading to increased invasion of peripheral bacteria and viruses as well as cells of the peripheral immune system, which will cause a defense response against these invaders (Blau et al., 2012; Marques et al., 2013). Moreover, increased clearance of damaged or misfolded proteins and other cellular debris becomes necessary with increasing age and these aggregates will activate the immune system (predominantly microglia) (Fonseca et al., 2004; Czirr and Wyss-Coray, 2012).

Of note, up-regulation of neuroinflammatory genes with aging clearly delivers an explanation why neurodegenerative diseases such as AD become increasingly abundant with advanced age. Based on our findings, we derive the hypothesis that disease-related mutations become only detrimental when the respective genes become expressed or activated. As such, aging is the strongest risk factor for AD (Lopez, 2011). The reason why late-onset AD (LOAD) is associated with increasing age is not yet fully understood. However, accumulating evidence and a plethora of genome-wide association studies (GWAS) link LOAD to a fast growing number of genetic risk factors and it seems feasible that these genetic risk factors result in LOAD due to changes at the transcriptome and proteome level. Indeed, in our hippocampal aging study in mice, we found up-regulation of several homologs, interaction partners or other closely related genes to almost all genes that appear to be the top 10 single genetic risk factors for LOAD as designated by the AlzGene database (Bertram et al., 2007). Of particular interest in light of our data is the gene coding for complement receptor 1 (CR1), since this receptor binds processed C4 and C3 complement proteins including the products of the *C4b* gene, which we found to be most prominently up-regulated in the aging hippocampus. Single nucleotide polymorphisms (SNPs) and copy number variations (CNVs) in the human CR1 gene were found to be highly associated with late onset AD in a number of studies and across ethnic groups (Bertram et al., 2007; Lambert et al., 2009; Biffi et al., 2010, 2012; Carrasquillo et al., 2010; Chibnik et al., 2011; Brouwers et al., 2012; Crehan et al., 2012; Hazrati et al., 2012; Keenan et al., 2012; Ma et al., 2014). This makes it very tempting to draw the conclusion that increased expression of C3 and C4 with aging will result in aberrant regulation of the complement cascade, which in turn lead to neurodegeneration in carriers of the “wrong” CR1 allele. Aberrant regulation could lead to neurodegeneration in two different, non-mutual exclusive ways. On the one hand, differential CR1 regulatory activity could lead to less effective Aβ aggregate clearance (Fonseca et al., 2004). On the other hand, de-regulated activation of the complement system could result in collateral damage by overactive inflammation (reviewed in Czirr and Wyss-Coray, 2012). Along this line, CR1 inhibition was shown to prevent microglia activation (Crehan et al., 2013). Of note, mice do not express CR1 but mice that lack the murine ortholog *Cr1-related protein Y* (*Crry*) show reduced inflammatory responses and attenuated increases in AD-related disease progress biomarkers (Killick et al., 2013), further suggesting that activation of the complement system is a key feature of the aging hippocampus that also plays a role in AD pathogenesis.

The exact mechanism of altered CR1 regulatory function in this complex network is still to be elucidated but a beneficial effect of complement inhibition in general has been demonstrated (Rancan et al., 2003; Fonseca et al., 2004; Leinhase et al., 2006; Kulkarni et al., 2008, 2011; Pillay et al., 2008), yet this data remains controversial (Wyss-Coray et al., 2002; Loeffler, 2004; Maier et al., 2008). Nevertheless, our data support the view that targeting the complement system and other inflammatory pathways poses an intriguing possibility for the treatment of aging-associated diseases and cognitive decline, which has recently been demonstrated for inhibition of NF-κB signaling in a mouse model of AD (Liu et al., 2014). Likewise, physical activity in rodents and humans has been shown to improve cognitive abilities during aging (van Praag et al., 1999; Praag et al., 2005; Erickson et al., 2011). Remarkably, a recent study could show in aged mice that voluntary wheel running leads to a reduction in C4b expression in the hippocampus (Kohman et al., 2011), demonstrating a clear correlation between C4 levels and cognitive abilities, which is further supported by the finding that C4 inhibition by external application of a vaccinia virus complement control protein has beneficial effects on memory performance in mouse models of AD (Kulkarni et al., 2008, 2011).

While it is not entirely clear if the age-associated induction of immune genes is cause or consequence of altered glia cell function that arise on the background of altered activity and/or increased cell number (Lolova, 1991; Amenta et al., 1998; Long et al., 1999; Blalock et al., 2003; Takahashi et al., 2006; Hayakawa et al., 2007) astrocytes and especially microglia are clearly good candidates for a potent source of immune system molecules (Hosokawa et al., 2003). We did not observe significant changes in the number of neurons, astrocytes or microglia in our study. In line with this, the corresponding marker genes for neurons and microglia were not altered in our gene-expression analysis. The astrocyte marker gene gfap was however increased during aging, indicating that the inflammatory response in our study in mainly linked to a change in the active state of glia cells. However, also neurons are known to secrete complement proteins (Shen et al., 1997; Terai et al., 1997; Hosokawa et al., 2003) and future studies should therefore also focus on untangling which cell types are the main contributors to the observed neuroinflammatory program. In any case *C4b* is an interesting gene, since its expression strongly correlated with age and it was in fact one of the most significant changes that have been reported by others and within this study. It was already found to be up-regulated as one of the very few DEGs in the comparison of hippocampal expression in 3-month-old mice with 16-month-old mice (3-fold up- regulation) (Peleg et al., 2010). Interestingly, out of the 12 up-regulated genes found in this independent study, 6 genes were also found up-regulated in the present study. Along with *C4b* and *C3*—another complement component frequently found up-regulated—these were 1700112E06Rik, BC061194, Cox8b and Sult1c2. Hence, these 6 genes obviously belong to a set of genes that are highly associated with murine hippocampal aging. In addition, other studies have observed complement-gene up-regulation in studies of aging in the hippocampus as well as in the prefrontal cortex (Verbitsky et al., 2004; Reichwald et al., 2009; Bordner et al., 2011; Stephan et al., 2013).

To our knowledge, the present study is the first to also specifically report the role of long-non-coding RNAs in the aging hippocampus. We found several long-non-coding RNAs to be differentially expressed. Little is known so far on the role of these RNAs, but the up-regulation of *Neat1* and the associated ncRNA *Malat1* was interesting since *Malat1* is highly abundant in neurons and has been associated with the regulation of gene expression and splicing, but was also found to affect the expression of genes linked to synaptic function (Bernard et al., 2010). Of note, a previous study reported that *Malat1* is increased in the hippocampus of alcoholics (Kryger et al., 2012), a condition clearly linked to cognitive impairment. Another interesting finding was the up-regulation of *Pisd-ps1*. Though the *Pisd-ps1* ncRNA has no annotated function so far, it has been described to be up-regulated with aging (Bordner et al., 2011; Sousa-Victor et al., 2014). *Pisd-ps1* also harbors a known RNA-editing site and was one of the few transcripts that show a higher editing frequency in 29-month-old mice. Thus, this ncRNA might be implicated in brain aging and warrants further research on its regulatory functions.

To obtain more insight on the mechanisms that drive age-associated changes in hippocampal gene expression, we analyzed the promoters of the differentially regulated transcripts. We found high enrichment of TFBS involved in pro-inflammatory signaling cascades. This trend was even more pronounced in 29-month-old mice, which supports our interpretation of increasing neuroinflammation with advancing age as part of the aging program. One of the key factors contributing to the neuroinflammatory gene expression response in the aging hippocampus was STAT1. Interestingly, mice that lack STAT1 show enhanced memory performance and are resistant to memory impairment induced by Aß peptides injected into the hippocampus (Hsu et al., 2014). Thus, activation of STAT1 might be a key step in age-associated memory impairment.

RNA sequencing also allows the analysis of differential splicing events and previous data suggest that differential exon usage undergoes substantial changes during brain development (Tollervey et al., 2011; Mazin et al., 2013). Differential splicing has also been observed in postmortem brain tissue from patients that suffered from sporadic AD (Twine et al., 2011; Mills et al., 2013) and in mouse models for amyloid deposition (Kim et al., 2012). This data is in line with increased protein levels of spliceosome components in AD patients (Bai et al., 2013). However, RNA sequencing has not been used to analyze changes in the aging hippocampus as described in this study. The first interesting observation is that massive changes in RNA splicing occur when we compared the hippocampus of 3-month-old mice to the 24- and 29-month groups. Another highly interesting observation was that there was little to no overlap among the genes differentially expressed and the genes that were differentially spliced during aging. The most striking finding is, however, that genes affected by age-associated changes in splicing were associated with neuronal and synaptic function, including neurite outgrowth and LTP. This is in line with the well-described decline in synaptic plasticity with aging (Blau et al., 2012). A number of interesting candidate genes were identified and await further analysis. For example, we identified *Spectrin β, non-erythrocytic 1* (*Sptbn1*) to be differentially spliced during aging. *Sptbn1* is involved in tethering the actin cytoskeleton to the plasma membrane, which is necessary for neurite outgrowth and possibly spine morphology and motility (Lee et al., 2012). Interestingly, introduction of the observed exon change in this gene may indicate a shift toward an isoform that cannot perform this function anymore, since it lacks the essential membrane-tethering PH protein domain. Together, these results confirm findings in the literature and make differential exon usage analysis a plausible tool for the study of age-related changes in the transcriptome. The fact that the age-associated inflammatory response is driven by changes in gene expression that could be linked to a number of key transcription factors while genes linked to synaptic plasticity are mainly affected by alternative splicing also suggests that functionally distinct gene expression programs in the adult brain are regulated by specific mechanisms.

In conclusion, our data deciphers at an unprecedented depth the hippocampal transcriptional program linked to aging. While changes in the absolute levels of RNA transcripts are linked to an inflammatory response and especially activation of the complement system, changes in alterative splicing affect genes linked to synaptic plasticity. At the same time RNA editing does not appear to play a major, genome-wide role in the aging hippocampus. This data provides important novel information, and multiple starting points for further analysis of potential therapeutic interventions and strongly suggests a causal relationship between aging-dependent changes in gene expression and the late onset of neurodegenerative diseases, most prominently AD.

### Conflict of interest statement

The authors declare that the research was conducted in the absence of any commercial or financial relationships that could be construed as a potential conflict of interest.

## Supplementary material

Figure S1 **Immunohistochemical analysis of hippocampal cell number. (A)** Representative images showing immunostaining for the Neuronal maker protein Neuronal N (NeuN), the Microglia marker Ionized calcium binding adaptor molecule 1 (IBA1) and the astrocyte marker *Glial fibrillary acidic protein* (GFAP) in 3 and 29-month old mice. **(B)** Quantification of A. CA1; hippocampal subregion CA1, cc; corpus calosum. Scale bar: 100 μm.

Figure S2 **Functionally enriched pathway in 3 vs. 24 and 29 month old mice identified on the basis of gene array data. Left panel**: Enriched pathways identified on the basis of differentially expressed genes comparing hippocampal RNA from 3- vs. 24-month-old mice. **Right panel**: Enriched pathways identified on the basis of differentially expressed genes comparing hippocampal RNA from 3 vs. 29-month-old mice.

Figure S3 **Genetic architecture and domain structure of Sptbn1**. The figure was downloaded from the ENSEMBLE database (version e76). It shows the genomic locus of Sptbn1 and its annotated transcribed isoforms including exon structure and encoded protein domains. Isoform 2 is marked in yellow. This was the isoform that is likely underlying the switch in exon usage detected in RNA-seq data in 24M and 29M groups.
