# Supplementary figures and images for: De-regulation of gene expression and alternative splicing affects distinct cellular pathways in the aging hippocampus
Source: Front Cell Neurosci. 2014 Nov 13;8:373. doi: 10.3389/fncel.2014.00373 (PMC4230043; doi:10.3389/fncel.2014.00373)

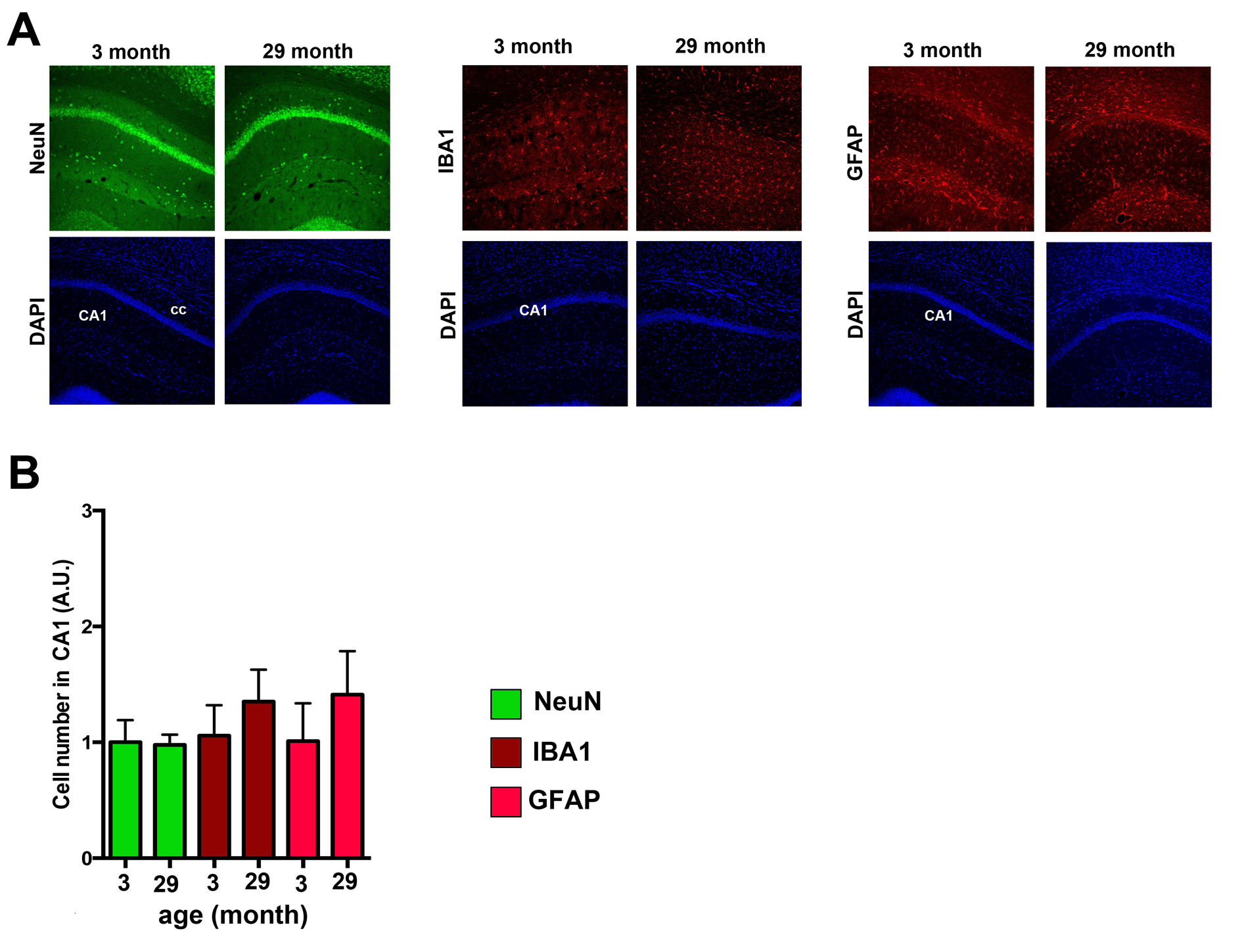

Supplement: Figure S1 — Immunohistochemical analysis of hippocampal cell number. (A) Representative images showing immunostaining for the Neuronal maker protein Neuronal N (NeuN), the Microglia marker Ionized calcium binding adaptor molecule 1 (IBA1) and the astrocyte marker Glial fibrillary acidic protein (GFAP) in 3 and 29-month old mice. (B) Quantification of A. CA1; hippocampal subregion CA1, cc; corpus calosum. Scale bar: 100 μm. [file Image1.JPEG]

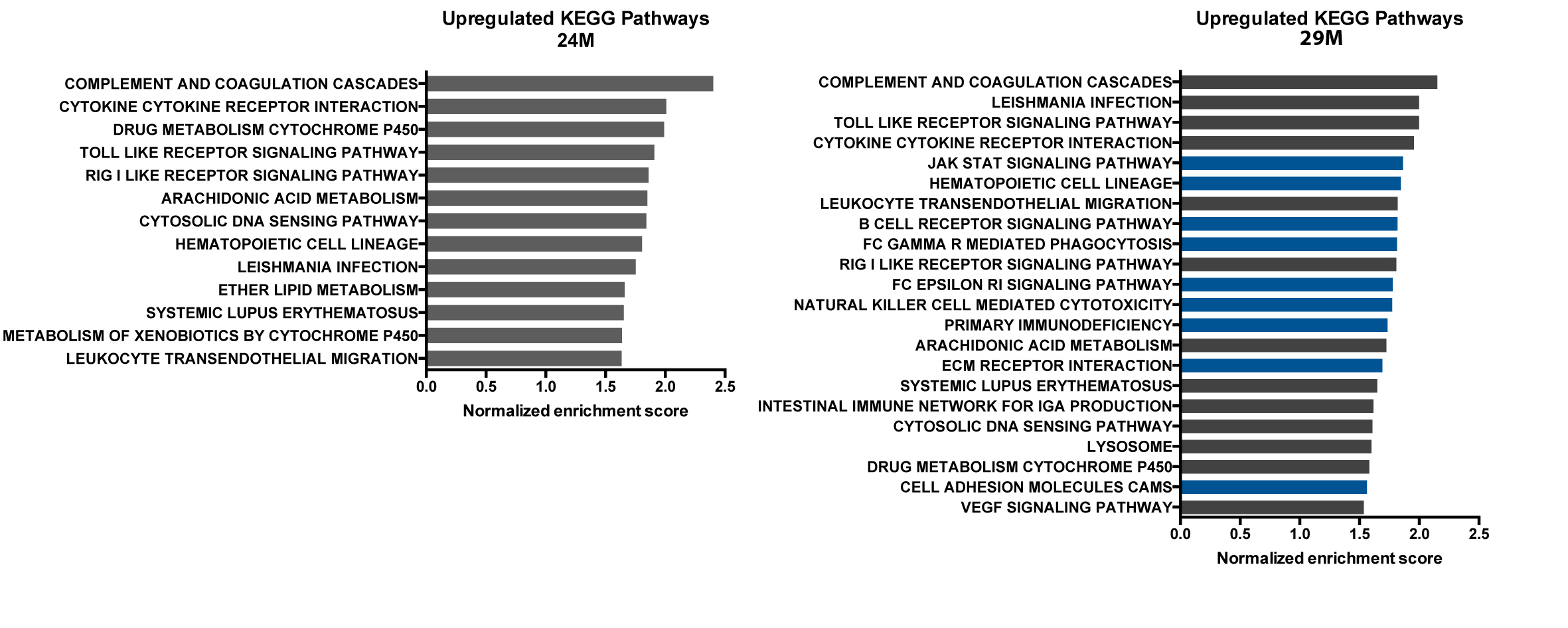

Supplement: Figure S2 — Functionally enriched pathway in 3 vs. 24 and 29 month old mice identified on the basis of gene array data. Left panel: Enriched pathways identified on the basis of differentially expressed genes comparing hippocampal RNA from 3- vs. 24-month-old mice. Right panel: Enriched pathways identified on the basis of differentially expressed genes comparing hippocampal RNA from 3 vs. 29-month-old mice. [file Image2.JPEG]

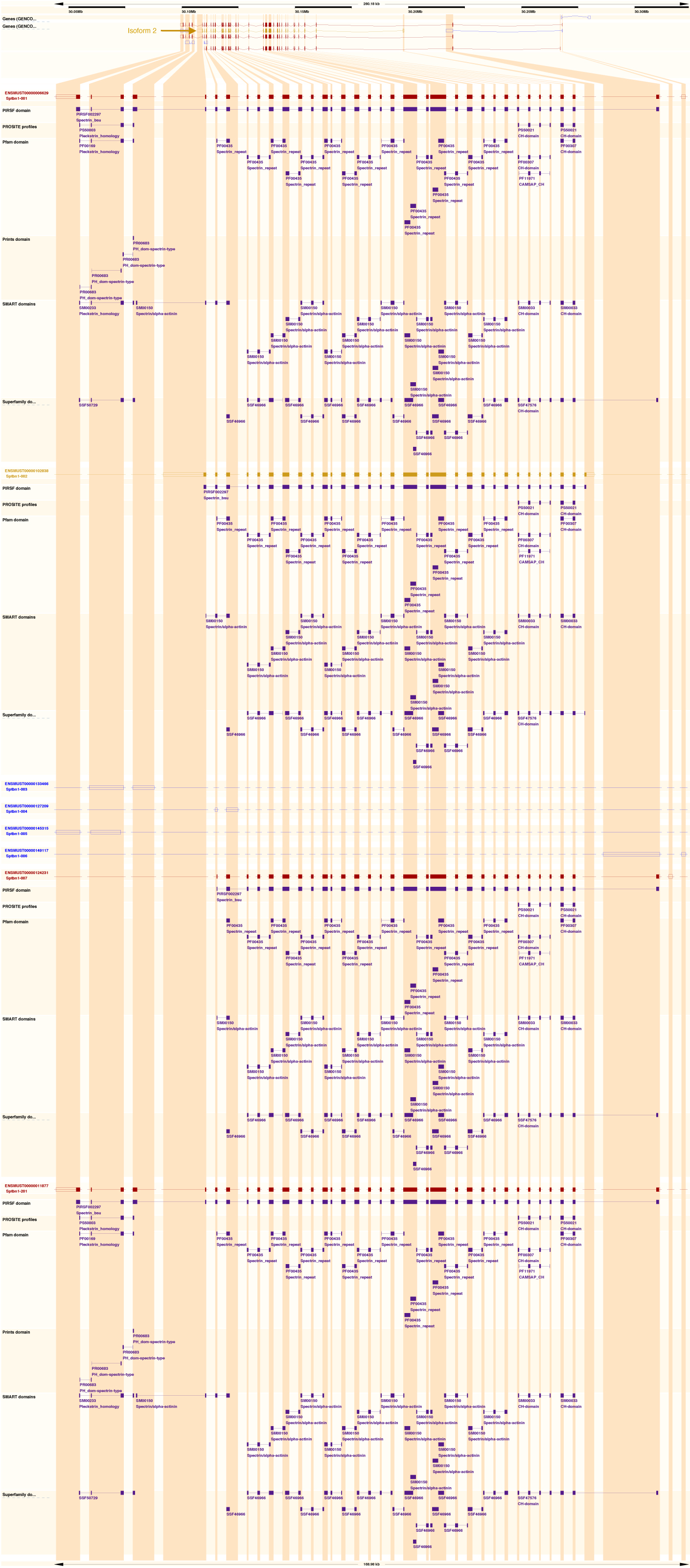

Supplement: Figure S3 — Genetic architecture and domain structure of Sptbn1. The figure was downloaded from the ENSEMBLE database (version e76). It shows the genomic locus of Sptbn1 and its annotated transcribed isoforms including exon structure and encoded protein domains. Isoform 2 is marked in yellow. This was the isoform that is likely underlying the switch in exon usage detected in RNA-seq data in 24M and 29M groups. [file Image3.JPEG]
